# The Biochemistry of *Vitreoscilla* hemoglobin

**DOI:** 10.5936/csbj.201210002

**Published:** 2012-10-29

**Authors:** Benjamin C. Stark, Kanak L. Dikshit, Krishna R. Pagilla

**Affiliations:** a Biology Division, Department of Biological and Chemical Sciences, Illinois Institute of Technology, Chicago IL 60616, USA; b Institute of Microbial Technology, Sec-39a, Chandigarh, 160036, India; c Department of Civil and Architectural Engineering, Illinois Institute of Technology, Chicago IL 60616, USA

## Abstract

The hemoglobin (VHb) from *Vitreoscilla* was the first bacterial hemoglobin discovered. Its structure and function have been extensively investigated, and engineering of a wide variety of heterologous organisms to express VHb has been performed to increase their growth and productivity. This strategy has shown promise in applications as far-ranging as the production of antibiotics and petrochemical replacements by microorganisms to increasing stress tolerance in plants. These applications of “VHb technology” have generally been of the “black box” variety, wherein the endpoint studied is an increase in the levels of a certain product or improved growth and survival. Their eventual optimization, however, will require a thorough understanding of the various functions and activities of VHb, and how VHb expression ripples to affect metabolism more generally. Here we review the current knowledge of these topics. VHb's functions all involve oxygen binding (and often delivery) in one way or another. Several biochemical and structure-function studies have provided an insight into the molecular details of this binding and delivery. VHb activities are varied. They include supply of oxygen to oxygenases and the respiratory chain, particularly under low oxygen conditions; oxygen sensing and modulation of transcription factor activity; and detoxification of NO, and seem to require interactions of VHb with “partner proteins”. VHb expression affects the levels of ATP and NADH, although not enormously. VHb expression may affect the level of many compounds of intermediary metabolism, and, apparently, alters the levels of expression of many genes. Thus, the metabolic changes in organisms engineered to express VHb are likely to be numerous and complicated.

## Introduction

In 1966 Dale Webster discovered the first bacterial hemoglobin, now known as VHb, in the Gram-negative *Vitreoscilla* sp. C1 [1]. Ironically, despite its being a soluble protein, but because of the close similarities of its spectral properties to those of the terminal respiratory oxidase (cytochrome *o*), this protein was initially identified as “soluble cytochrome *o*”. In 1969 Appleby provided evidence for a hemoglobin in *Rhizobium*, but this protein was not fully characterized [2]. It was not until the amino acid sequence of VHb was determined in 1986, that the real nature of VHb as a hemoglobin rather than a soluble cytochrome was apparent [3]. Although it was fairly clear from VHb's amino acid sequence that it was comprised of a single globin domain without additional structural elements, solution of its crystal structure [4] confirmed that the three-dimensional structure of VHb adheres remarkably closely to the classic globin fold.

In the years since 1986 biochemical, and especially genome mining studies, have shown that hemoglobins are widespread among microbes. It was also discovered that bacterial hemoglobins fall into three distinct structural categories [5]. One of these is the single globin domain (“single domain”) structure exhibited by VHb. The second group (“flavohemoglobins”) is comprised of single subunit proteins in which a globin domain highly homologous to that of VHb is fused to a flavin-binding (reductase) domain. The third group (“truncated hemoglobins”) contains hemoglobins that are considerably smaller than VHb and share a “two over two alpha helix structure”; this group can be further classified into three sub-categories [6]. Truncated hemoglobins are found in bacteria, fungi and plants, but not Archaea [6, 7]. In addition to bacteria, flavohemoglobins can be found in lower eukaryotes [8]. Both the flavohemoglobins and truncated hemoglobins are fairly common, but the single domain hemoglobins are rarer, so far being found in only a handful of bacteria [8]. Two hemoglobins (“protoglobins”) related to single domain bacterial globins, but with extra amino acids at both termini have been identified in Archaea [9].

The functions of the bacterial hemoglobins, although all involving oxygen binding in some way, appear to be varied. The main role of the flavohemoglobins appears to be to detoxify NO by converting it to NO_3_^−^, presumably using both O_2_ binding by the globin domain and reduction via the flavin-binding domain [10, 11]. A recently characterized flavohemoglobin is able also to oxidize lactate to pyruvate [12]. The functions of the truncated hemoglobins have not been conclusively determined, but may include scavenging oxygen to protect the nitrogen fixing system, and detoxification of NO by its conversion to nitrate [6, 7].

The single (globin) domain VHb has been the best studied of all the bacterial hemoglobins, and probably has a number of functions. Perhaps its main role is binding of oxygen at low concentrations and its direct delivery to the terminal respiratory oxidase(s) (e.g., cytochrome *o*) [13–15]. This function fits well with VHb's induction at low oxygen [16, 17] and the biology of its native host (*Vitreoscilla*), which despite being an obligate aerobe, inhabits hypoxic habitats [18]. VHb may have other functions as well, including delivery of oxygen to oxygenases [19], detoxification of NO by its conversion to nitrate [20], and sensing oxygen concentrations and transmitting this information to affect the activity of transcription factors [21]. The latter function is presumably related to the correlation, found in one study using recombinant *E. coli*, of VHb presence with changes in transcription of hundreds of genes [22]. These topics will be discussed in more detail below.

## Cloning of the VHb encoding gene, *vgb*

The gene (*vgb*) encoding VHb was cloned in 1988 [23, 24]. This allowed detailed examination of transcriptional control of *vgb*, use of site-directed mutagenesis to explore VHb structure-function questions, and, particularly using various strains of recombinant *E. coli* engineered to express VHb, investigation of how VHb functions as well as the effects it has on metabolism more generally. It also allowed, beginning in 1990 [25], genetic engineering with *vgb* of what has now become a large group of prokaryotic, lower eukaryotic, and higher plant heterologous hosts to improve their usefulness (reviewed in [8, 26, 27]). Some of these studies will be discussed below.

## Oxygen binding properties of VHb

Following the correct identification of VHb as a hemoglobin, efforts switched to investigate its role as an oxygen binding protein. It was initially proposed that, under oxygen limiting conditions, VHb is induced in order to bind the remaining oxygen and deliver it to the terminal respiratory oxidase(s) to maintain aerobic respiration at a high level under these conditions [28]. Consistent with this role evidence was presented that, in *E. coli* engineered with *vgb*, much of the VHb (about 40%) is in the periplasm [29]. Ramandeep et al. [13], however, provided evidence that the periplasmic location of VHb may be an artifact, and that VHb appears instead to be concentrated, in both native *Vitreoscilla* and *E. coli* expressing VHb, in the cytoplasm just below the plasma membrane, where, as would be the case for a periplasmic location, it would allow it to interact with and deliver O_2_ to the terminal respiratory oxidases.

A role for VHb in oxygen delivery to cytochrome *o* was consistent with a report in 1996 [30] indicating that VHb expressed in *E. coli* increased the apparent affinity of cytochrome *o* for oxygen by about 75%, but had no effect on the oxygen affinity of cytochrome *d*. Eventually direct evidence was provided to support this function. Ramandeep et al. [13] showed that VHb binds specifically to respiratory membranes and to cytochrome *o* incorporated into lipid vesicles, and then Park et al. [14] showed through two hybrid screen experiments that there is a direct binding of VHb to the catalytic, oxygen binding subunit of the cytochrome *o*'s from *Vitreoscilla*, *E. coli*, and *Pseudomonas aeruginosa*. A subsequent two hybrid screen experiment showed that VHb also binds to the catalytic subunit of the alternative, low oxygen induced, high oxygen affinity terminal respiratory oxidase, cytochrome *d* from *E. coli* [15].

Equilibrium binding studies measured the dissociation constant of VHb for oxygen at about 6 µM [28, 31]. Orii and Webster [32] used laser flash photolysis to measure both *k*_*on*_ and *k*_*off*_ for oxygen binding to VHb; *k*_*on*_ was not remarkable compared to those for other oxygen binding proteins, but *k*_*off*_ was unusually large [28]. Structurally, this may be due to features of the binding pocket of VHb that will be discussed below. Functionally, this may be a feature of VHb that optimizes its ability to deliver oxygen to various partner proteins (e.g., cytochrome *o*, oxygenases). Interestingly, the oxygen dissociation constant calculated from the ratio of *k*_*off*_ to *k*_*on*_ was 12 times greater than that measured by the equilibrium binding studies.

## Mutants of VHb: Structure-function studies

The oxygen binding properties and abilities of VHb to interact with other cellular partners can be correlated with three structural features of the protein [4]. One of these is the structural organization of its proximal and distal heme sites, which are characterized by lack of a proper E helix and E7 gate for the entry and exit of ligands. A second is lack of a defined structure in the D region between the C and E helices, which imparts higher structural flexibility to this region. Third is a 90° rotation of the imidazole ring of the proximal histidine at F8. This results in a unique hydrogen bonding network involving Fe-HisF8-GluH23-TyrG5 that determines the redox potential and the electronic structure of the heme iron. A series of VHb mutants, with alterations in the distal and proximal heme sites and the D region, have been generated that provided valuable structure-function information on the role of key residues within these portions of the protein (Table 1).

**Table 1:** Structure-function studies using site-directed mutants of VHb.

VHb region/site altered	effect(s)	reference
Distal heme site		[31]
GlnE7àLeuE7	no effect on oxygen or CO binding	
GlnE7àHisE7	no stable oxygenated form seen,* binding with CO slowed	

Distal heme site		[33]
GlnE7àHisE7	Oxygen affinity increases 2.6X,* changes to CO, CN^−^ binding	
ProE8àAlaE8	Oxygen affinity unchanged, changes to CO, CN^−^ binding	
GlnE7àHisE7, ProE8àAlaE8	No deoxygenation possible, changes to CO, CN^−^ binding	

Last part of D region and first part of E region		[35]
AspD5àAlaD5	Altered CO binding, normal heme content, impaired binding to DNT dioxygenase	
GlyD7àIleD7	∼Normal CO binding, normal heme content	
ArgE1àAsnE1	Altered CO binding, low heme content, ∼normal binding to DNT dioxygenase	
GlnE2àAlaE2	Altered CO binding, low heme content, ∼normal binding to DNT dioxygenase	
GluE3àGlnE3	∼Normal CO-binding, low heme content, impaired binding to DNT dioxygenase	
LeuE5àGlyE5	∼Normal CO binding, normal heme content,
	∼Normal binding to DNT dioxygenase	

proximal heme site (TyrG5, TyrH12)		[34]
TyrG5àPheG5	Altered CO binding, NO dioxygenase activity nearly eliminated, unable to form stable oxyform	
TyrG5àLeuG5	Altered CO binding, NO dioxygenase activity nearly eliminated, unable to form stable oxyform	
TyrH12àPheH12	Slightly altered CO binding, increased NO dioxygenase activity, affinity for oxygen decreased 2-3X	
TyrH12àLeuH12	Slightly altered CO binding, increased NO dioxygenase activity, affinity for oxygen decreased 2-3X	

* The reasons for the differences in the two studies with the same mutant are not known.

Structural and ligand binding studies on distal site mutants of VHb confirmed that residues E7-E10 in this region do not adopt an alpha helical conformation, preventing GlnE7 from performing the conventional function of ligand stabilization at the active site [31, 33]. These studies also suggested that the mechanisms of entry, stabilization and exit of ligands within the active site of VHb may be distinct from those of conventional Hbs. Subsequent studies on other VHb mutants demonstrated that the proximal site interactions involving TyrG5 contribute significantly to the structural integrity and function of VHb [34]. As mentioned above, the D region of VHb, connecting helices C and E is disordered and highly flexible. Biophysical and ligand binding studies on site directed mutants of VHb indicated that the D region is involved in maintaining the heme-globin stoichiometry and interactions with partner proteins, in this case the flavin domain of 2,4-DNT dioxygenase from *Burkholderia* [35]. The structural flexibility of the D region may allow VHb to interact with various partner proteins, in turn allowing it to be involved in a variety of cellular functions.

## Other functions of VHb

In addition to the apparent direct binding of VHb to terminal respiratory oxidases, presumably to deliver oxygen and enhance respiration under conditions of low aeration, it appears that VHb has a number of other functions, many of which also appear to involve its interaction with other proteins. These are summarized in Table 2.

**Table 2:** Interactions of VHb with partner proteins

interacting partner protein	Possible function(s)	reference
*Vitreoscilla* cytochrome *o*	Enhanced respiration at low oxygen	[13]
*Vitreoscilla*, *E. coli*, *P. aeruginosa* cytochrome *o* subunit I	Enhanced respiration at low oxygen	[14]
*Burkholderia* strain DNT, DNT dioxygenase	Delivery of oxygen to oxygenase	[19]
*E. coli* flavohemoglobin flavoreductase domain	NO-dioxygenation	[20]
*E. coli* cytochrome *d*	Enhanced respiration at low oxygen	[15]
*E. coli* (transcription factors) Fnr, OxyR	Modulation of redox state	[21]

### Function of VHb as an alternative terminal oxidase

An *E. coli* mutant strain lacking both terminal oxidases (i.e., both cytochromes *o* and *d*) and thus unable to grow aerobically was engineered to express VHb [36]. The resulting strain was able to grow aerobically. In addition, purified VHb added to membranes of the engineered double mutant resulted in a 5X increase in respiration, and this increase was nearly eliminated by the addition of KCN. These results indicate that VHb can itself act as an alternative respiratory terminal oxidase.

### The VHb interactome

Although VHb exists predominantly as a homodimer, it has also been found to exist in a monomeric state under some physiological conditions [20]. As mentioned above, it has been speculated that the unique structural organization of VHb and the apparent conformational flexibility of the D region may allow it to interact with different partners and perform multiple cellular functions. An NADH-met reductase co-purifies along with VHb in *Vitreoscilla* [37, 38] and may be required to keep it in the functional ferrous state in the native host to facilitate oxygen delivery to the respiring membrane. As mentioned above, a major role of VHb seems to be to deliver oxygen to the terminal respiratory oxidases under conditions of limiting aeration. In particular VHb can interact specifically with subunit I of cytochrome *o* and the A subunit of cytochrome *d*, which in each case is the site of oxygen binding and reduction in respiration [14, 15]. Although the molecular mechanisms of the VHb interactions with cytochromes *o* and *d* are not fully understood, these studies indicate its involvement in supporting cellular respiration in a unique way.

Construction of a chimeric gene in which a flavoreductase domain encoding region is fused with *vgb* results in a flavoHb like hybrid protein that interacts with nitric oxide and relieves the toxicity of nitrosative stress in *E. coli* [20]. The same study also indicated that VHb itself may interact with an independent flavoreductase protein for this function. In addition, there is evidence that VHb may be able to deliver oxygen directly to oxygenases involved in aerobic catabolism of aromatic compounds [19]; this is discussed in more detail below. VHb also displays a high propensity for lipid binding that results in a several fold decrease in its oxygen affinity and increase in its oxygen release [39]. This opens the possibility that interactions of VHb with other non-protein cellular components may modulate its function in diverse ways.

### VHb and control of gene expression

A recent study demonstrated that the redox state of the cell mediates interactions of VHb with the global transcription regulators, OxyR and Fnr, which in turn may affect metabolism in a broad way [21]. These and perhaps similar, yet undiscovered regulatory functions for VHb may be reflected in the results of a microarray study on *E. coli* expressing VHb. These results indicated that the presence of VHb brings about significant changes in expression of hundreds of genes, including those in almost every category of cellular function [22]. Isarankura-Na-Ayudhya et. al. [40] also found that VHb expression in *E. coli* could affect the levels of proteins in several different metabolic categories. Another study found a five fold increase in the level of cytochrome *o* and 1.5 fold increase in the level of cytochrome *d* in VHb expressing *E. coli* under microaerobic conditions [30]. Effects of VHb expression on specific aspects of metabolism have been the focus of a number of other studies; these are detailed in the following section.

## Metabolic changes correlated with VHb expression

Table 3 summarizes the effects on various parts of metabolism that have been correlated with VHb expression. Even before the correct identification of VHb as a hemoglobin, some aspects of its activities and effects on metabolism were investigated in its native host, *Vitreoscilla*. One of these is related to the induction of VHb synthesis by low aeration conditions [16]. Eventually, this induction was found to occur at the transcriptional level [17]. In *E. coli* *vgb* appears to be transcriptionally controlled by at least three oxygen responsive transcription factors (ArcA, Fnr, and OxyR) as well as Crp [21, 41–43]. By a mechanism which is as yet undetermined, the induction in VHb levels is coordinated with a large (50-fold) increase in heme biosynthesis, to supply each VHb dimer with its two necessary heme *b* groups [16].

**Table 3:** Effects on metabolism correlated with VHb expression/presence

organism	metabolic property	VHb-correlated effect	reference
*Vitreoscilla*	Hydrogen peroxide production	VHb has hydrogen peroxide producing activity	[44]
*Vitreoscilla*	superoxide metabolism	VHb can convert superoxide to O_2_	[45]
*E. coli*	ATP	no increase in [ATP] or transmembrane DpH, but increases in ATP synthesis, flux	[46]
*E. coli*	ATP, H+/O, transmembrane DpH	2X increase in [ATP]; 50% increase in H^+^/O; 60% increase in transmembrane DpH	[47]
*E. coli*	ATP, NADH flux	modeling suggests 13% increase in ATP flux; 62% increase in NADH flux	[48]
*E. coli*	NAD(P)H	1.8X decrease in [NAD(P)H], 2.4X decrease in net generation of NAD(P)H	[49]
*E. coli*	ATP	∼ 60% increase in [ATP]	[50]
*E. coli*	ATP, NAD, NADH (time courses during growth)	∼ 50% decrease in [ATP], similar [NADH] in log phase; similar [ATP] and ∼ 50% decrease in [NADH] in stationary phase	[51]
*E. coli*	acid production	no effect	[52]
*E. coli*	acetate, ethanol, formate, lactate, succinate, CO_2_	25-72% reductions	[48]
*E. coli*	acetate, ethanol, formate, lactate, succinate, CO_2_	variable effects (increases, decreases, ∼ no change)	[50]
*S. cerevisiaea*	ethanol	∼ 30% increase	[53]
*E. coli*	ethanol	increases of ∼ 10-300%	[51, 69]
*Serratia marcescens*	acetoin, butanediol, acid	lower acetoin and butanediol, higher acid	[54]
*Enterobacter aerogenes*	acetoin, butanediol	∼ 50%% increase for acetoin; ∼ 80% increase for butanediol	[55]
*Burkholderia* strain DNT	DNT dioxygenase	(*in vitro*) enhancement (2.7X) by added VHb	[19]
*Burkholderia* strain DNT	2-chlorobenzoate degradation pathway	change from meta pathway to pathway for complete dechlorination; nearly two-fold increase in affinity for 2-CBA	[60, 61]

It was also found early on that VHb has hydrogen peroxide synthesizing activity [44] and may be able, in its oxidized (Fe^+3^) form, to detoxify superoxide by the reaction: VHb(Fe^+3^) + O_2_^−^ → VHb(Fe^+2^) + O_2_ [45].

### VHb and ATP and NADH levels

The proposed (and eventually substantiated; see above) role for VHb in enhancing aerobic respiration was also investigated from the standpoint of VHb-correlated changes in cellular ATP and NADH levels, the hypothesis being that ATP levels might increase and NADH levels decrease due to enhanced respiration. Chen and Bailey [46] found no increase in ATP levels or transmembrane ΔpH in VHb-expressing *E. coli* compared to VHb-negative controls but the faster growth rate of the VHb strain and an additional NMR study suggested increases in ATP synthesis of 68% and ATP flux of 30% due to VHb expression. An additional study comparing the same two strains, however, indicated an increase in ATP level of about two-fold, as well as increases of 50-60% in H^+^/O and transmembrane ΔpH that were correlated with VHb expression [47].

Tsai et al. [48] used measurements of a number of metabolites (but not direct measurement of either ATP or NADH) to model ATP and NADH flux, again in *E. coli* expressing VHb compared to the VHb-free control strain. The model suggested a modest increase (17%) in ATP flux and a somewhat larger increase (56%) in NADH flux correlated with VHb expression. NAD(P)H levels and rate of net generation of NAD(P)H were again investigated in VHb-expressing *E. coli* and in this case, compared to the VHb-free control strain, these parameters decreased by 1.8-fold and 2.4-fold, respectively [49]. A subsequent study, again using VHb-expressing *E. coli*, found an approximately 60% increase in ATP levels associated with VHb presence [50].

In the experiments described above essentially only a single stage in the cultures, for the most part either late log/early stationary phase or what can be inferred to have been stationary phase, was sampled. A more recent study of ATP and NADH/NAD+ levels, again in *E. coli* engineered to express VHb and compared against the matched control strain without VHb, was made from log through stationary phases [51]. Levels of ATP, NAD^+^, and NADH decreased by large amounts in both VHb^+^ and VHb^−^ cells from log through stationary phases. There was a tendency for (roughly two-fold) higher ATP levels in the VHb^−^ cells in log phase but nearly identical ATP levels for both strains in stationary phase. The NADH levels of the two strains were similar in log phase, but that of the VHb^−^ cells was about twice that of the VHb^+^ cells in stationary phase.

Taken together the measurements that have been made on ATP and NADH levels in recombinant *E. coli* expressing VHb compared with those in VHb^−^ cells are not completely consistent, but do indicate that the presence of VHb does not have large effects on NADH and ATP levels. The most consistent result is that VHb is correlated with an about 50% decrease in NADH levels. This corresponds to the apparent role of VHb in enhancing aerobic respiration, in which case greater amounts of NADH would be fed into the electron transport chain. In the case of ATP levels, it may be that ATP synthesis is increased somewhat due to VHb and its attendant enhancement of aerobic oxidative phosphorylation, and that the added ATP is used to result in faster growth and increased production of certain metabolites, leading to a steady state ATP level similar to VHb^−^ cells [46, 51].

### VHb and intermediary metabolism

The effects of VHb expression on metabolism more generally has been investigated to some degree, usually using recombinant *E. coli*. An early study [52] showed that expression of VHb in *E. coli* did not alter the rate of acid synthesis. Two later studies [48, 50] measured production of acetate, ethanol, formate, D-lactate, succinate, and CO_2_ by matched strains of *E. coli*, one expressing and one not expressing VHb, under conditions of low oxygen. Although carbon balance was met for both strains in both studies, the results were very different. In [48], VHb was correlated with a lowered production of all products (by 25-72%) and lowered excreted pyruvate (by up to 3-fold). In [50] VHb was correlated with an ∼ 60% increase in CO_2_ production, with sizable increases in ethanol and lactate production, a slight increase in succinate production, and sizable decreases in production of only acetate and formate.

In [48] the measured data were incorporated into a model of a portion of *E. coli* metabolism (glycolysis, the TCA cycle, the pentose phosphate shunt, and mixed acid fermentation). The model suggested that the presence of VHb resulted in a shift of carbon input away from the TCA cycle to the pentose phosphate pathway. The study by Frey et al. [50] also used the measured data in a carbon flux model and concluded that VHb results in increased flux through glycolysis and branching of the TCA cycle. Because the actual measurements of metabolites in the two studies were so different, however, a firm picture of how VHb presence changes these portions of metabolism is not yet available.

Simpler analyses, looking at the effects of VHb expression on levels of one or a few metabolites have been performed on several engineered microbes. *Saccharomyces cerevisiae* engineered with VHb showed an apparent increase of about 30% in ethanol production, but the results were complicated by the fact that the ethanol measurements were made in stationary phase for the VHb-expressing strain and late log phase for the VHb^−^ strain [53]. Wei et al. [54] engineered *Serratia marcescens* to express VHb and measured its effects on fermentation (total acid, acetoin, and butanediol production). The effects were growth medium dependent, but the most consistent occurred in LB supplemented with glucose, in which VHb was correlated with lower acetoin and butanediol production as well as much higher acid accumulation in stationary phase. A similar study in *Enterobacter aerogenes* [55] showed that VHb expression was correlated with increases of ∼ 50-80% in acetoin and butanediol production.

### VHb and aerobic metabolism of aromatic compounds

In the process of investigating the positive effects of VHb expression in heterologous bacterial hosts to improve a variety of useful properties, several bioremediating bacteria (*Pseudomonas*, *Xanthomonas*, and *Burkholderia* species) were engineered to express VHb [56-64; reviewed in 65,66]. Increases of 15-73% in (aerobic) biodegradation of benzoic acid, dinitrotoluene and 2-chlorobenzoic acid were correlated with VHb expression. It is presumed that VHb-mediated delivery of oxygen to the respiratory chain, as described above, may be responsible for at least some of this enhancement.

Direct delivery of oxygen to the oxygenases involved in aromatic metabolism may also be involved. The first enzyme in the 2,4-dinitrotoluene degradation pathway of *Burkholderia* strain DNT (DNT dioxygenase) was expressed in *E. coli* both with and without VHb, VHb being correlated with a 3-4 fold increase in the activity of the enzyme in whole cell extracts [67]. Addition of purified VHb to partially purified DNT dioxygenase *in vitro* resulted in a 2.7 fold increase in dioxygenase activity compared to reactions without added VHb [19]. The latter experiment is most easily interpreted if VHb interacts directly with the dioxygenase (as a partner protein; see also above) to deliver molecular oxygen to it.

Experiments in which expression of VHb in *Burkholderia* strain DNT enhanced degradation of dinitrotoluene and 2-chlorobenzoate (2-CBA) provided details of the effects of VHb on physiology and biochemistry. So et al. [63] found that *Burkholderia* strain DNT expressing VHb (strain YV1) had an oxygen uptake rate and affinity for carbon substrate that were about twice those of the matched strain not expressing VHb (strain DNT). A similar (about two-fold) increase in affinity for 2-CBA was seen for strain YV1 compared to strain DNT [61].

Perhaps more interesting are indications from experiments on the same pair of strains metabolizing 2-CBA; in these experiments VHb was correlated with the choice of catabolic pathway by which 2-CBA was metabolized [60]. Under low aeration conditions, where the effects of VHb are expected to be most apparent, strain DNT appeared to metabolize 2-CBA by a mixture of pathways that resulted in a minority of the 2-CBA being mineralized and apparent accumulation of chlorine containing intermediates that then inhibited further 2-CBA metabolism. Under the same conditions strain YV1 utilized pathway(s) that resulted in mineralization of essentially all of the 2-CBA and avoided production of the inhibitory compounds. It is possible that this effect is related to either a general increase in oxygen supply to the cells mediated by VHb, or a preference of VHb for oxygen delivery to particular oxygenases.

### VHb and ethanol production

Two recent studies using an ethanologenic strain of *E. coli* (strain FBR5 [68]) examined the effects of VHb on ethanol production in a variety of growth media (LB supplemented with glucose, xylose, or corn fiber hydrolysate) and growth conditions [51, 69]. Strain FBR5 was engineered at the Fermentation Biochemistry Research Unit of the National Center for Agricultural Utilization Research, Peoria, IL; this strain has been mutated so that all naturally occurring fermentation pathways have been blocked, and then transformed with the ethanol pathway from *Zymomonas mobilis* encoded on plasmid pLOI297 [70] so that it produces only ethanol by fermentation. FBR5 was transformed with plasmid pTS3, which is compatible with pLOI297 and contains *vgb* in a construct that results in a moderate level of VHb expression [69]. This VHb level was correlated with increases in ethanol production that were found consistently across a variety of growth media and conditions, although the amount of the increase was variable (a range of approximately 10-300%) and medium (LB supplemented with either xylose, glucose, or corn fiber hydrolysate) and condition (shake flasks versus fermenters; aerobic versus limited aeration) dependent.

The increase in ethanol correlated with VHb expression appears to be the biological correlate of results from several groups that have investigated the effects on the production of 2,3-butanediol and ethanol resulting from supplying cultures (mechanically) with small amounts of oxygen. Jansen et al. [71] found optimal 2,3-butanediol productivity by *Klebsiella oxytoca* at an oxygen supply greater than zero, while Okuda et al. [72] and Nieves et al. [73] found ethanol production by ethanologenic *E. coli* to be increased by about 25% by addition of small amounts of air to the cultures. Thus, the effect of VHb on enhancing ethanol levels is presumed to be related to the increase in oxygen supply it provides to cells, although the details of what that mechanism might be are as yet unknown.

## Summary and outlook

It is clear that genetic engineering using *vgb*/VHb can be used to enhance biological production of a variety of potentially valuable compounds by microbes and even the growth and robustness of higher plants. It is also clear that the optimization of such applications will require a comprehensive understanding of how expression of VHb affects physiology and metabolism. Although much work has been done to try to determine these effects, because of the fragmentary nature of the investigations and variability of results in some cases, such a comprehensive understanding has not yet been achieved.

What is known is that the presence of VHb seems to affect steady state concentrations and rates of production of ATP and NADH modestly, and also may affect production of fermentation products, oxygen uptake in some cases, and affinity of cells for various substrates. In the case of 2-CBA metabolism by *Burkholderia* strain DNT, VHb seems to be involved in the choice of catabolic pathways. The details of how VHb affects physiology and metabolism likely involve, at least in some cases, its direct interaction with partner proteins, examples of which were discussed above. These interactions, in turn, must be related to VHb structure, both in its oxygen binding site where oxygen binding and delivery properties are determined, as well as elements such as the D region, which may provide a flexible binding site for multiple partners.

Future studies that are able to coordinate VHb structure-function data, information on interaction of VHb with partner proteins, and more robust information regarding how VHb affects metabolic pathways will undoubtedly lead to design of improved systems in which VHb expression is used to enhance valuable biological processes.
